# Gene expression profiling of cuticular proteins across the moult cycle of the crab *Portunus pelagicus*

**DOI:** 10.1186/1741-7007-5-45

**Published:** 2007-10-10

**Authors:** Anna V Kuballa, David J Merritt, Abigail Elizur

**Affiliations:** 1 Department of Primary Industries and Fisheries (DPI&F), Animal Science, Bribie Island, Queensland 4507, Australia; 2 School of Integrative Biology, The University of Queensland, St Lucia, Queensland 4072, Australia; 3 Faculty of Science, Health and Education, University of the Sunshine Coast, Maroochydore, Queensland 4558, Australia

## Abstract

**Background:**

Crustaceans represent an attractive model to study biomineralization and cuticle matrix formation, as these events are precisely timed to occur at certain stages of the moult cycle. Moulting, the process by which crustaceans shed their exoskeleton, involves the partial breakdown of the old exoskeleton and the synthesis of a new cuticle. This cuticle is subdivided into layers, some of which become calcified while others remain uncalcified. The cuticle matrix consists of many different proteins that confer the physical properties, such as pliability, of the exoskeleton.

**Results:**

We have used a custom cDNA microarray chip, developed for the blue swimmer crab *Portunus pelagicus*, to generate expression profiles of genes involved in exoskeletal formation across the moult cycle. A total of 21 distinct moult-cycle related differentially expressed transcripts representing crustacean cuticular proteins were isolated. Of these, 13 contained copies of the cuticle_1 domain previously isolated from calcified regions of the crustacean exoskeleton, four transcripts contained a chitin_bind_4 domain (RR consensus sequence) associated with both the calcified and un-calcified cuticle of crustaceans, and four transcripts contained an unannotated domain (PfamB_109992) previously isolated from *C. pagurus*. Additionally, cryptocyanin, a hemolymph protein involved in cuticle synthesis and structural integrity, also displays differential expression related to the moult cycle. Moult stage-specific expression analysis of these transcripts revealed that differential gene expression occurs both among transcripts containing the same domain and among transcripts containing different domains.

**Conclusion:**

The large variety of genes associated with cuticle formation, and their differential expression across the crustacean moult cycle, point to the complexity of the processes associated with cuticle formation and hardening. This study provides a molecular entry path into the investigation of the gene networks associated with cuticle formation.

## Background

Arthropods, such as arachnids, crustaceans and insects, represent the most abundant phylum in the animal kingdom. They are common throughout marine, freshwater, terrestrial, and aerial environments. The success of arthropods can partly be attributed to the protection offered by their characteristic tough exoskeleton. With the advantage of this protective armour, however, comes the problem of growth restriction; arthropods overcome this through periodic moulting, or shedding of the exoskeleton.

The crustacean moult cycle is divided into four discrete stages; pre-moult, ecdysis, post-moult, and intermoult [1], based on the morphology of the exoskeleton. During pre-moult the underlying epidermis separates from the old cuticle (apolysis), the old cuticle is partially digested and reabsorbed, and new epi- and exo-cuticle are secreted. The old exoskeleton is shed during ecdysis. After ecdysis, during the post-moult stage, expansion of the partially formed new exoskeleton occurs followed by the tanning and mineralisation of the pre-ecdysial layers, and the deposition and hardening of the endocuticle. At the intermoult stage a mature, fully developed exoskeleton is formed [2].

The crustacean intermoult integument consists of four layers. The thin outermost layer, the epicuticle, is characterised by an absence of chitin and is principally composed of proteins, lipids and calcium salts. Beneath it lies the exocuticle, composed of chitin as well as proteins and calcium salts. The epi- and exo-cuticle are both secreted prior to ecdysis. The post-ecdysial endocuticle is divided into two layers, the principle layer consisting of proteins, chitin and calcium salts, and the membranous layer that remains un-calcified [3-5]. Table 1 summarises the features of arthropod integumental layers.

**Table 1:** Summary of arthropod cuticle strata subdivisions and characteristics (modified from [4])

	**Insects**	**Crustaceans**		
	**Cuticle layers**	**Cuticle layers**	**Organic phase**	**Mineral phase**

Pre-ecdysial layers	Epicuticle	Epicuticle	Lipoproteic	Calcite
	Exocuticle	Exocuticle	Chitin-protein	Calcite
Post-ecdysial layers (Endocuticle)	Endocuticle (with mesocuticle)	Principle layer	Chitin-protein	Calcite
		Membranous layer	Chitin-protein	--

The exoskeleton of crustaceans and insects is formed by cells of the hypodermis, an epithelial layer located beneath the cuticle, during both the pre- and post-moult stages [5,6]. At the beginning of pre-moult, the hypodermis secretes a moulting fluid containing enzymes for digestion of the inner layers of the old cuticle. Secretion of a crustacean's new exoskeleton by the hypodermal cells begins during pre-moult, before the old exoskeleton is shed [2,7]. Formation of the new exoskeleton occurs in layers. The thin epicuticle is formed first, secreted into the extracellular space between the epidermis and old exoskeleton, and a thicker exocuticle layer then appears beneath the new epicuticle during late pre-moult. At pre-moult the developing integument has invaginations over the entire surface of the animal. This increases the surface area of the new exoskeleton within the restricted space of the old exoskeleton. Ecdysis enables the expansion of the new exoskeleton [8]. Immediately after ecdysis, while the new exoskeleton is still soft, unfolding and expanding to a larger size than the old exoskeleton, the crab continues secreting the endocuticle layer beneath the new exocuticle. Mineralisation of the new exoskeleton is initiated after moulting [9]. The endocuticle layer continues to increase in thickness, while calcification and sclerotization of the new exoskeleton progresses.

The hypodermis synthesizes almost all of the proteins in the cuticle, however, hemocytes and several hemolymph proteins also contribute to the synthesis of the new exoskeleton [10]. Crustacean cryptocyanin, a member of the hemocyanin gene family, has been implicated in the transport of hormones, phenols, and some cuticular proteins to the hypodermis. Cryptocyanin itself may also be used directly as a structural component of the new exoskeleton [11-14].

The organic matrix of the crustacean cuticle is a complex structure composed mainly of α-chitin microfibrils embedded in a protein matrix [8]. The protein matrices contain a large number of proteins with different properties. These proteins can be grouped according to the type of domain that they possess. One domain type, the chitin_bind_4 domain (Pfam nomenclature), containing the Rebers-Riddiford (RR) consensus sequence, has chitin binding properties [15]. While another, the cuticle_1 domain (Pfam nomenclature), is thought to be associated with calcification as it has been found in proteins isolated from the calcified regions of the crustacean cuticle [16,17].

Many genes that are involved in the formation of the new exoskeleton have been isolated both from insects and crustaceans. However, despite extensive research, the molecular events associated with cuticle formation in crustaceans still remain poorly understood. To investigate and gain a better understanding of this phenomenon, we set out to identify genes associated with cuticle formation, and to study their expression patterns throughout the moult cycle of the blue swimmer crab *Portunus pelagicus*. We used microarray technology, which provides a powerful, holistic approach to study gene expression in relation to changing physiological states.

## Results

### Microarray expression analysis

**Figure 1:**
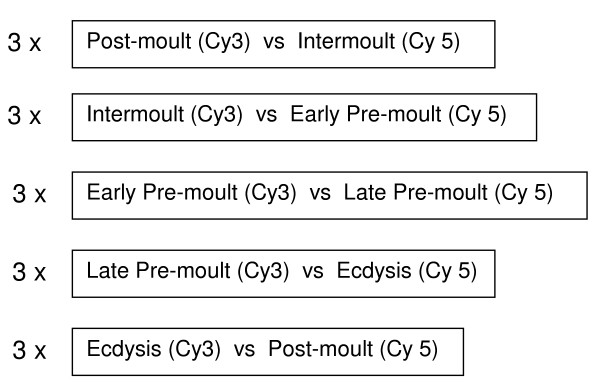
Experimental design for moult stage hybridisation.

Custom *P. pelagicus *cDNA arrays were created using transcripts isolated from both whole crabs, and the brain, eyestalk, mandibular organ (MO) and Y-organ of crabs in the following five moult cycle stages: post-moult, intermoult, early pre-moult, late pre-moult, and ecdysis. Dual channel cDNA microarray hybridisation experiments, using RNA isolated from *P. pelagicus *crabs in the above mentioned stages, were used to identify differentially expressed genes across consecutive stages of the moult cycle. Figure 1 shows a depiction of the hybridisation experiments comparing each moult cycle stage. Many transcripts were identified within the scope of the microarray experiments described, however only those associated directly with cuticle formation will be discussed here.

### Nomenclature of differentially expressed cuticle protein transcripts

Cluster and subsequent BLAST analysis revealed 21 unique, differentially expressed transcripts encoding arthropod cuticle proteins. These were screened in Pfam, and were each found to contain one of four different domain types: cuticle_1, chitin_bind_4, Pfam B 109992, and CBM 14. A total of 13 unique cDNAs, each displaying differential expression profiles relating to moult stage, contain the cuticle_1 domain. Four distinct differentially expressed cDNAs contain the chitin_bind_4 domain (a variant of the RR consensus sequence). Additionally, four different cDNAs contain a domain associated with cuticle protein CPCP1876 of the rock crab *Cancer pagurus *(protein accession number P81584), tentatively termed the Pfam B 109992 domain. Furthermore one transcript containing the CBM 14 domain also known as the Peritrophin-A domain (found in chitin binding proteins [18]) was also differentially expressed. The common factor linking these transcripts lies in the domain type they contain, however, they do not appear to be encoded by the same gene nor code for the same protein. Three transcripts of an additional type of cuticular protein that displayed sequence homology to a LDLa domain containing chitin binding protein from *Drosophila *(NP_730442), termed *vermiform*, also displayed moult cycle-related differential expression. Apart from the cuticle proteins, cryptocyanin, a hemolymph protein known to be involved in cuticle formation, also displayed differential expression across the moult stages. These transcripts have been named according to the type of protein domain identified in the sequence, and numbered consecutively (Table 2).

**Table 2:** Guide to the nomenclature of the cuticle associated proteins described (numbers indicate unique transcripts with the same domain type)

**Domain type**	**Nomenclature**
*P. pelagicus *cuticle_1 (transcripts 1 – 13)	PpCUT1-13
*P. pelagicus *chitin_bind_4 (transcripts 1 – 4)	PpCB1-4
*P. pelagicus *PfamB_109992 (transcripts 1 – 4)	PpBD1-4
*P. pelagicus *CBM 14	PpCBM
*P. pelagicus *vermiform like (transcripts 1 – 3)	PpVER1-3
*P. pelagicus *cryptocyanin (transcripts 1 and 2)	PpCRYP1 and 2

The temporal differential expression patterns for genes relating specifically to cuticle formation are summarised in Tables 3, 4, 5, 6, 7 (where multiple values for the same transcript indicate expression levels for different cDNA probes on the microarray that make up a contig), listed according to moult stage. The heat map in Table 8 summarises the differential expression profiles of transcripts related to cuticle formation isolated in this study.

**Table 3:** The list of cuticle associated proteins up-regulated in post-moult (Cy3) crabs when compared against crabs in the intermoult stage (Cy5)

**Transcript Ids (accession no.)**	**Gene IDs (BLAST results)**	**Score (bits)**	**E value**	* M	*t	*P value	*Adjusted P value
PpCUT12 (GenBank: EF102004)	P81580 CUPC1 Cuticle protein CP1158 CPCP1158 *Cancer pagurus*	70.1	6e-11	4.949	5.485	0.002	0.036
				4.867	5.669	0.002	0.033
				4.277	5.495	0.002	0.036
				4.26	5.313	0.003	0.04
				4.233	5.532	0.002	0.035
				4.02	5.129	0.003	0.045
				3.929	5.259	0.003	0.041
				3.844	5.262	0.003	0.041
PpBD2 (GenBank: EF102014)	DQ288154 *Callinectes sapidus *calcified cuticle protein CP15.0 mRNA	745	0.0	4.471	9.086	0	0.007
				3.709	6.694	0.001	0.019
	ABB91679 calcified cuticle protein CP15.0 *C. sapidus*	214	2e-54	3.414	6.097	0.001	0.028
PpCUT13 (GenBank: EF102005)	P81580 CUPC1 Cuticle protein CP1158 (CPCP1158) *C. pagurus*	69.7	6e-11	4.415	5.963	0.002	0.029
				4.397	5.489	0.002	0.036
				4.161	5.83	0.002	0.031
				2.104	7.01	0.001	0.017
PpCBM (GenBank: EF102017)	AAR06266 peritrophic membrane chitin binding protein 2 *Trichoplusia ni *(Cabbage lopper) AAD40313 chitinase 1 *Penaeus monodon*	55.8	6e-07	3.13	5.858	0.002	0.031
		46.6	3e-04				
PpVER3 (GenBank: EF102020)	NP_730443 LDLa domain containing chitin binding protein 1 CG8756-PB, isoform B *Drosophila melanogaster*	110	5e-23	3.024	8.459	0	0.009
PpBD1 (GenBank: EF102013)	DQ288154 *C. sapidus *calcified cuticle protein CP15.0 mRNA	87.7	3e-14	2.817	4.966	0.004	0.05
				2.811	5.293	0.003	0.041
	P81584 CUPC5 Cuticle protein CP1876 CPCP1876 *C. pagurus*	145	1e-33	2.754	2.754	0.003	0.041
PpBD3 (GenBank: EF102015)	P81584 CUPC5 Cuticle protein CP1876 CPCP1876 *C. pagurus*	80.1	1e-13	2.687	5.321	0.003	0.04
PpVER1 (GenBank: EF102018)	NP_730442 LDLa domain containing chitin binding protein 1 CG8756-PA *Drosophila*	155	1e-38	2.609	8.689	0	0.008
PpBD4 (GenBank: EF102016)	P81584 CUPC5 Cuticle protein CP1876 CPCP1876 *C. pagurus*	53.5	2e-06	1.823	5.729	0.002	0.032

M = *log*_2 _fold change in expression, t = *t*-statistic, and the P value = *t*-distribution. The adjusted P values were calculated using the false discovery rate (FDR) procedure. Positive values of M and t indicate up-regulation in the Cy3 sample (* multiple values for the same transcript indicate expression levels for different cDNA probes on the microarray that make up a contig).

**Table 4:** The list of cuticle associated proteins down-regulated in post-moult stage (Cy3) crabs when compared against crabs in the intermoult (Cy5)

**Transcript Ids (Accession no.)**	**Gene IDs (BLAST results)**	**Score (bits)**	**E value**	*M	*t	*P value	*Adjusted P value
PpCRYP1 (GenBank: EF102021)	AF091261 *Cancer magister *cryptocyanin (CC1) mRNA	517	2e-143	-5.519	-17.319	0	0.002
				-5.366	-19.323	0	0.002
	ABB59714 cryptocyanin 2 *C. magister*	572	8e-162	-5.16	-20.243	0	0.002
				-4.971	-18.46	0	0.002
				-4.966	-12.183	0	0.003
				-4.948	-11.536	0	0.003
				-4.885	-19.268	0	0.002
				-4.841	-16.889	0	0.002
				-4.821	-17.521	0	0.002
				-4.817	-17.824	0	0.002
				-4.79	-18.98	0	0.002
				-4.787	-15.616	0	0.002
				-4.785	-17.019	0	0.002
				-4.785	-17.095	0	0.002
				-4.572	-17.015	0	0.002
				-4.493	-15.838	0	0.002
				-4.392	-14.665	0	0.002
				-4.183	-7.188	0.001	0.015
				-3.427	-8.404	0	0.009
PpCRYP2 (GenBank: EF102022)	DQ230982 *C. magister *cryptocyanin 2 (Cc2) AAD09762 cryptocyanin *C. magister*	157	7e-35	-5.153	-17.813	0	0.002
		226	8e-68	-4.759	-14.431	0	0.002
PpCUT10 (GenBank: EF102002)	P81585 CUPC6 Cuticle protein CP434 (CPCP434) *C. pagurus*	76.6	3e-13	-3.626	-11.234	0	0.004
PpCUT9 (GenBank: EF102001)	P81580 CUPC1 Cuticle protein CP1158 (CPCP1158) *C. pagurus*	100	5e-20	-3.176	-11.079	0	0.004
PpCUT7 (GenBank: EF101999)	P81580 CUPC1 Cuticle protein CP1158 (CPCP1158) *C. pagurus*	80.9	7e-14	-2.865	-8.21	0	0.01
PpCUT8 (GenBank: EF102000)	P81580 CUPC1 Cuticle protein CP1158 (CPCP1158) *C. pagurus*	142	2e-32	-2.556	-9.073	0	0.007

M = *log*_2 _fold change in expression, t = *t*-statistic, and the P value = *t*-distribution. The adjusted P values were calculated using the false discovery rate (FDR) procedure. Negative values of M and t indicate down-regulation in the Cy3 sample (* multiple values for the same transcript indicate expression levels for different cDNA probes on the microarray that make up a contig).

**Table 5:** The list of cuticle associated proteins, in order of expression levels, that are up-regulated in the intermoult stage (Cy3) when compared against early pre-moult crabs (Cy5)

**Transcript IDs (accession no.)**	**Gene IDs (BLAST results)**	**Score (bits)**	**E value**	*M	*t	*P value	*Adjusted P value
PpCUT1 (GenBank: EF101993)	P81582 CUPC3 Cuticle protein CP1246 (CPCP1246) *C. pagurus*	148	3e-34	7.715	12.071	0	0.027
PpCUT2 (GenBank: EF101994)	P81582 CUPC3 Cuticle protein CP1246 (CPCP1246) *C. pagurus*	143	9e-33	7.397	15.388	0	0.015
PpCB1 (GenBank: EF102006)	DQ288153 *C. sapidus *calcified cuticle protein CP6.1	135	1e-28	7.109	14.424	0	0.018
	ABB91678 calcified cuticle protein CP6.1 *C. sapidus*	83.2	3e-15				
PpCB2 (GenBank: EF102007)	DQ288153 *C. sapidus *calcified cuticle protein CP6.1 mRNA	61.9	5e-06	7.089	15.538	0	0.015
	ABB91678 calcified cuticle protein CP6.1 *C. sapidus*	65.5	5e-09				
PpBD1 (GenBank: EF102013)	DQ288154 *C. sapidus *calcified cuticle protein CP15.0 mRNA	87.7	3e-14	6.44	14.605	0	0.017
	P81584 CUPC5 Cuticle protein CP1876 CPCP1876 *C. pagurus*	145	1e-33	6.249	11.706	0	0.029
PpCUT3 (GenBank: EF101995)	P81588 CUC10 Cuticle protein CP498 (CPCP498) *C. pagurus*	54.3	2e-06	6.121	19.272	0	0.012
				5.898	19.173	0	0.012
PpCB3 (GenBank: EF102008)	DQ288153 *C. sapidus *calcified cuticle protein CP6.1 mRNA	54.0	3e-04	5.995	13.013	0	0.022
	ABB91678 calcified cuticle protein CP6.1 *C. sapidus*	79.7	4e-14				
PpBD2 [GenBank: EF102014]	DQ288154 *C. sapidus *calcified cuticle protein CP15.0 mRNA	745	0.0	5.327	23.449	0	0.012
	ABB91679 calcified cuticle protein CP15.0 *C. sapidus*	214	2e-54				
PpCUT4 (GenBank: EF101996)	P81582 CUPC3 Cuticle protein CP1246 (CPCP1246) *C. pagurus*	42.0	3e-08	5.274	17.593	0	0.014
PpCB4 (GenBank: EF102009)	AY752734 *C. sapidus *arthrodial cuticle protein AMP6.0 mRNA	559	3e-156	5.176	20.083	0	0.012
	AAV28477 arthrodial cuticle protein AMP6.0 *C. sapidus*	192	5e-48	4.616	20.25	0	0.012
PpCUT5 (GenBank: EF101997)	P81580 CUPC1 Cuticle protein CP1158 (CPCP1158) *C. pagurus*	155	3e-36	4.718	12.398	0	0.025
PpCUT6 (GenBank: EF101998)	P81587 CUPC8 Cuticle protein CP463 (CPCP463) *C. pagurus*	81.6	4e-14	4.26	11.666	0	0.029
PpCUT7 (GenBank: EF101999)	P81580 CUPC1 Cuticle protein CP1158 (CPCP1158) *C. pagurus*	80.9	7e-14	3.418	19.596	0	0.012
PpCUT8 (GenBank: EF102000)	P81580 CUPC1 Cuticle protein CP1158 (CPCP1158) *C. pagurus*	142	2e-32	3.351	18.96	0	0.012
PpCUT11 (GenBank: EF102003)	P81580 CUPC1 Cuticle protein CP1158 (CPCP1158) *C. pagurus*	41.2	0.003	3.192	15.938	0	0.015
PpCUT10 (GenBank: EF102002)	P81585 CUPC6 Cuticle protein CP434 (CPCP434) *C. pagurus*	76.6	3e-13	3.041	17.14	0	0.015
PpCUT9 (GenBank: EF102001)	P81580 CUPC1 Cuticle protein CP1158 (CPCP1158) *C. pagurus*	100	5e-20	2.963	15.21	0	0.015
PpVER1 (GenBank: EF102018)	NP_730442 LDLa domain containing chitin binding protein 1 CG8756-PA *Drosophila*	155	1e-38	2.585	10.905	0	0.034

M = *log*_2 _fold change in expression, t = *t*-statistic, and the P value *t*-distribution. The adjusted P values were calculated using the false discovery rate (FDR) procedure. Positive values of M and t indicate up-regulation in the Cy3 sample (* multiple values for the same transcript indicate expression levels for different cDNA probes on the microarray that make up a contig).

**Table 6:** The list of cuticle associated proteins up-regulated in the late pre-moult (Cy3) stage when compared against crabs in ecdysis (Cy5)

**Transcript IDs (accession no.)**	**Gene IDs (BLAST results)**	**Score (bits)**	**E value**	*M	*t	*P value	*Adjusted P value
PpCRYP1 (GenBank: EF102021)	AF091261 *Cancer magister *cryptocyanin (CC1) ABB59714 cryptocyanin 2 *C. magister*	517	2e-143	5.334	20.137	0	0.01
		572	8e-162	5.07	19.923	0	0.01
				5.061	19.374	0	0.01
				4.875	16.555	0	0.01
				4.842	17.141	0	0.01
				4.747	15.569	0	0.01
				4.682	13.94	0	0.01
				4.536	17.265	0	0.01
				4.502	12.822	0	0.01
				4.453	12.362	0	0.01
				4.413	12.648	0	0.01
				4.39	12.755	0	0.01
				4.374	15.933	0	0.01
				4.354	10.194	0	0.015
				4.311	13.787	0	0.01
				4.179	10.362	0	0.015
				3.937	8.784	0	0.022
PpCRYP2 (GenBank: EF102022)	DQ230982 *C. magister *cryptocyanin 2 (Cc2) AAD09762 cryptocyanin *C. magister*	157	7e-35	4.873	16.433	0	0.01
		226	8e-68	4.304	11.836	0	0.011

M = *log*_2 _fold change in expression, t = *t*-statistic, and the P value *t*-distribution. The adjusted P values were calculated using the false discovery rate (FDR) procedure. Positive values of M and t indicate up-regulation in the Cy3 sample (* multiple values for the same transcript indicate expression levels for different cDNA probes on the microarray that make up a contig).

**Table 7:** The list of cuticle associated proteins down-regulated in crabs in the late pre-moult stage (Cy3) when compared against crabs at ecdysis (Cy5)

**Transcript IDs (accession no.)**	**Gene IDs (BLAST results)**	**Score (bits)**	**E value**	*M	*t	*P value	*Adjusted P value
PpCUT12 (GenBank: EF102004)	P81580 CUPC1 Cuticle protein CP1158 CPCP1158 *C. pagurus*	70.1	6e-11	-4.78	-6.756	0.001	0.044
				-3.931	-6.565	0.002	0.047
				-3.843	-7.666	0.001	0.03
				-3.739	-6.428	0.002	0.048
				-3.46	-6.36	0.002	0.049
				-3.034	-6.734	0.002	0.044
PpCUT1 (GenBank: EF101993)	P81582 CUPC3 Cuticle protein CP1246 (CPCP1246) *C. pagurus*	148	3e-34	-4.731	-6.517	0.002	0.048
PpCB1 (GenBank: EF102006)	DQ288153 *C. sapidus *calcified cuticle protein CP6.1 mRNA	135	1e-28	-4.198	-15.618	0	0.01
	ABB91678 calcified cuticle protein CP6.1 *C. sapidus*	83.2	3e-15				
PpCB3 (GenBank: EF102008)	DQ288153 *C. sapidus *calcified cuticle protein CP6.1 mRNA	54.0	3e-04	-3.956	-7.798	0.001	0.029
	ABB91678 calcified cuticle protein CP6.1 *C. sapidus*	79.7	4e-14				
PpCUT5 (GenBank: EF102010)	P81580 CUPC1 Cuticle protein CP1158 (CPCP1158) *C. pagurus*	155	3e-36	-3.674	-6.567	0.002	0.047
PpCUT11 (GenBank: EF102003)	P81580 CUPC1 Cuticle protein CP1158 (CPCP1158) *C. pagurus*	41.2	0.003	-2.631	-9.379	0	0.018
PpCUT9 (GenBank: EF102001)	P81580 CUPC1 Cuticle protein CP1158 (CPCP1158) *C. pagurus*	100	5e-20	-2.482	-7.892	0.001	0.028
PpCUT4 (GenBank: EF101996)	P81582 CUPC3 Cuticle protein CP1246 (CPCP1246) *C. pagurus*	42.0	3e-08	-1.64	-6.397	0.002	0.048
PpVER2 (GenBank: EF102019)	NP_730443 LDLa domain containing chitin binding protein 1 *Drosophila*	32.0	8.4	-2.676	-9.202	0	0.018

M = *log*_2 _fold change in expression, t = *t*-statistic, and the P value = *t*-distribution. The adjusted P values were calculated using the false discovery rate (FDR) procedure. Negative values of M and t indicate down-regulation in the Cy3 sample (* multiple values for the same transcript indicate expression levels for different cDNA probes on the microarray that make up a contig).

**Table 8:** Heat map of the differential expression profile for each moult stage

**List of genes**	**Post (Cy3) vs inter (Cy5)**	**Inter (Cy3) vs early pre (Cy5)**	**Early pre (Cy3) vs late pre (Cy5)**	**Late pre (Cy3) vs ecdysis (Cy5)**	**Ecdysis (Cy3) vs post (Cy5)**
PpVER2				↓	
PpCUT12	↑			↓	
PpCUT1		↑		↓	
PpCUT4		↑		↓	
PpCUT5		↑		↓	
PpCUT11		↑		↓	
PpCB1		↑		↓	
PpCB3		↑		↓	
PpCUT9	↓	↑		↓	
PpCUT7	↓	↑			
PpCUT8	↓	↑			
PpCUT10	↓	↑			
Cryptocyanin	↓			↑	
PpCUT2		↑			
PpCUT3		↑			
PpCUT6		↑			
PpCB2		↑			
PpCB4		↑			
PpBD1	↑	↑			
PpBD2	↑	↑			
PpVER1	↑	↑			
PpCUT13	↑				
PpBD3	↑				
PpBD4	↑				
PpCBM	↑				
PpVER3	↑				

Up arrow denotes up-regulation in the Cy3 channel, down arrow denotes down-regulation in the Cy3 channel.

### Post-moult (Cy3) vs intermoult (Cy5)

The transcripts related to cuticle formation that were up-regulated in the post-moult stage when compared against the intermoult stage, are documented in Table 3. Two transcripts containing the cuticle_1 domain, PpCUT12 and PpCUT13, were up-regulated in the post-moult stage. Each consisted of several cDNAs grouped together to form a contig. They were up-regulated by an average of 8.6- and 7.6-fold respectively. Four unique transcripts possessing the PfamB_109992 domain (PpBD1-4) were also up-regulated in the post-moult stage, they displayed a combined average up-regulation of 6.4-fold, and represent all of the PfamB_109992 domain-containing transcripts isolated in this study. Two of these transcripts were also up-regulated in intermoult when compared to pre-moult. No differential expression of transcripts containing the chitin_bind_4 domain was observed in the comparison between the post-moult and intermoult stages of the moult cycle.

The transcripts that display temporal down-regulation in the post-moult stage, when compared with intermoult, are listed in Table 4. Four different transcripts containing the cuticle_1 binding domain (PpCUT7-10) were down-regulated in the post-moult stage; they display a combined average of 6.2-fold down-regulation. These transcripts were also up-regulated in intermoult when compared to early pre-moult. Furthermore, PpCUT9 was also down-regulated in late pre-moult when compared to ecdysis. Two cryptocyanin transcripts (PpCRYP1 and 2) were highly down-regulated in the post-moult stage when compared to intermoult. Both transcripts consist of several cDNAs grouped together to form contigs, the combined average down-regulation for cryptocyanin was 9.6-fold.

### Intermoult (Cy3) vs early pre-moult (Cy5)

The up-regulated transcripts involved in cuticle formation that were observed for the intermoult stage when compared against early pre-moult, are listed in Table 5. Briefly, 11 unique cDNAs, all containing the cuticle_1 domain (PpCUT1-11), were up-regulated (by a combined average of 9.6-fold) in intermoult. This constitutes the highest number of cuticle_1 domain transcripts up-regulated in any of the moult stages. Four cDNAs containing the chitin_bind_4 (PpCB1-4) domain, were up-regulated at a combined average of 12-fold in intermoult. This is also the highest number of chitin_bind_4 domain transcripts up-regulated in any moult stage. Additionally, two transcripts containing the PfamB_109992 domain (PpBD1 and 2), were up-regulated in the intermoult stage, the combined average up-regulation for these was 12-fold.

No temporal down-regulation of transcripts involved in cuticle formation was observed in intermoult when compared to the early pre-moult stage.

### Early pre-moult (Cy3) vs late pre-moult (Cy5)

Microarray analysis indicates that no statistically significant differential gene expression can be observed between the early pre-moult and late pre-moult stages.

### Late pre-moult (Cy3) vs ecdysis (Cy5)

Two distinct cryptocyanin transcripts, each containing several cDNAs that aligned to form a contig, were up-regulated in late pre-moult when compared to ecdysis. The average up-regulation of both cryptocyanin transcripts in the late pre-moult stage was 9.2-fold. Table 6 displays the level of up-regulation for each transcript.

Six cuticle_1 domain-containing transcripts (PpCUT1, 4, 5, 9, 11 and 12) were down-regulated in late pre-moult when compared to ecdysis (combined average of sevenfold). Five of these cuticle_1 transcripts were up-regulated in the intermoult stage of the moult cycle when compared to early pre-moult. Two transcripts containing the chitin_bind_4 domain, PpCB1 and PpCB3, were also down-regulated in late pre-moult with a combined average down-regulation of 8.2-fold. Both of these chitin_bind_4 transcripts were up-regulated in intermoult when compared to early pre-moult. All of the transcripts that display temporal down-regulation in late pre-moult when compared to ecdysis are listed in Table 7.

### Ecdysis (Cy3) vs post-moult (Cy5)

Microarray analysis found no statistically significant differential gene expression between ecdysis and the post-moult stage of the moult cycle.

### Additional transcripts related to cuticle formation

Additional transcripts that displayed homology to other previously isolated cuticle proteins, also containing the above mentioned domains, were identified in this study. These transcripts, however, failed the stringent statistical analysis and cannot be defined as differentially expressed. The details and accession numbers for these transcripts are documented in Table 9.

**Table 9:** List of transcripts identified to be relevant to cuticle formation in crustaceans

**Transcript IDs**	**GenBank accession no.**	**Gene IDs (BLAST results)**	**Score (bits)**	**E value**	**Protein domain**
PpCB5	EF102010	AAV28478 calcified cuticle protein CP8.5 *C. sapidus*	113	4e-24	Chitin_bind_4
		BAC81566 calcification-associated peptide-1 *Procambarus clarkii*	84.7	2e-15	
		BAD16776 calcification-associated peptide-2 *P. clarkii*	53.5	4e-06	
		BAB13739 crustocalcin *Marsupenaeus japonicus*	42.7	0.007	
PpCB6	EF102011	EAT39443 cuticle protein, putative *Aedes aegypti*	78.2	7e-13	Chitin_bind_4
PpCB7	EF102012	P82119 CUO6_BLACR Cuticle protein 6 (BcNCP14.9) cockroach (domain match)	75.1	4e-12	Chitin_bind_4
49-12		P81582 CUPC3 Cuticle protein CP1246 *C. pagurus*	145	2e-33	Cuticle_1 (3)
3–61		AAR06266 peritrophic membrane chitin binding protein 2 *Trichoplusia ni *(cabbage looper)	43.9	0.003	CBM_14
6–13		P81590 CUPC9 Cuticle protein CP466 (CPCP466)	58.9	4e-07	Cuticle_1 (1)

These transcripts were not found to be differentially expressed under the stringent analysis conditions applied to the microarray data.

## Discussion

Moulting is an important biological process in arthropods as it is essential for growth, metamorphosis and reproduction. The formation of the new exoskeleton is integral to the moulting process. Although many proteins involved in cuticle synthesis and structural integrity have been previously isolated, little is known about the expression profiles of genes related to cuticle formation across the entire moult cycle. The objective of the current study was to use a *P. pelagicus *cDNA microarray developed in our laboratory to identify genes (both previously identified and new) that are involved in the formation of the crustacean exoskeleton. The microarrays were produced from 5000 cDNAs expressed in both the entire animal and in specific organs such as the brain, eyestalk, MO and Y-organ from all moult cycle stages. Thus the arrays were designed to study global gene expression profiles of genes relevant to the moulting process, across all moult stages. Microarray technology offers the potential to examine the expression patterns of many genes simultaneously, thus gaining a better understanding of gene function, interaction, and regulation.

Crustacean cuticles are composed of chitin rods embedded in a protein matrix [4], the physical properties of which depend, among other things, on the sequence of the constituent proteins and the extent of mineralisation [19]. Many of the proteins associated specifically with the cuticular matrix of crustaceans can be divided into groups, based on the type of domain that they contain. One group contains a cuticle_1 domain (Pfam); proteins with this domain have previously been isolated from the hard, calcified cuticle of crustaceans [16,17]. The restricted occurrence of this domain in calcified crustacean cuticle led to the suggestion that it could be involved in the calcification process, either as a nucleation factor for crystal formation or in regulating the growth and size of the calcium carbonate crystals once they have been formed [16]. We have isolated and profiled the differential expression of 13 unique transcripts containing such a domain across the moult cycle of *P. pelagicus*. Another group contains the RR consensus sequence (chitin_bind_4 in Pfam), demonstrated to be involved in chitin binding [15]. Proteins with chitin_bind_4 domains have previously been isolated from both calcified and un-calcified crustacean cuticle [16,20], and also from the cuticles of insects [21,22]. In the present study, four transcripts with the chitin_bind_4 domain were found to be differentially expressed across the *P. pelagicus *moult cycle. We have also isolated four unique, differentially expressed transcripts containing a domain that as yet remains unannotated, but has previously been isolated from the cuticle of *C. pagurus *[16], tentatively termed PfamB_109992 (Pfam). It is likely that the different domain types found in cuticle proteins are functionally relevant to the cuticles in which they occur. The expression of cryptocyanin was also found to be moult-cycle related. Unlike the other cuticle proteins, cryptocyanin is a hemolymph protein secreted by the hepatopancreas that has also been implicated in the formation of post-ecdysial cuticle [14].

**Figure 2:**
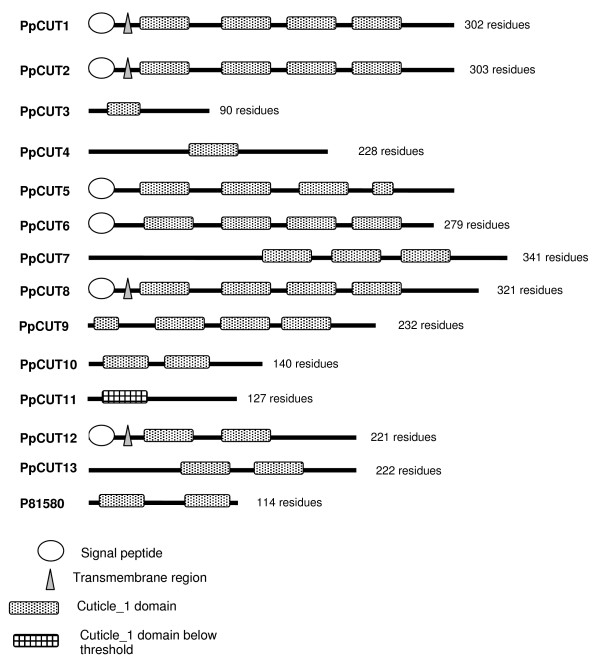
**Schematic diagram of amino acid sequences containing one or more cuticle_1 domains, including a cuticle protein previously isolated from *C. pagurus***. Note that the sequences derived from this study (PpCUT1-13) may represent partial sequences, the absence of a signal peptide in some transcripts, therefore, may or may not indicate an actual lack of signal peptide. The length of the amino acid sequence is denoted by the number of residues.

**Figure 3:**
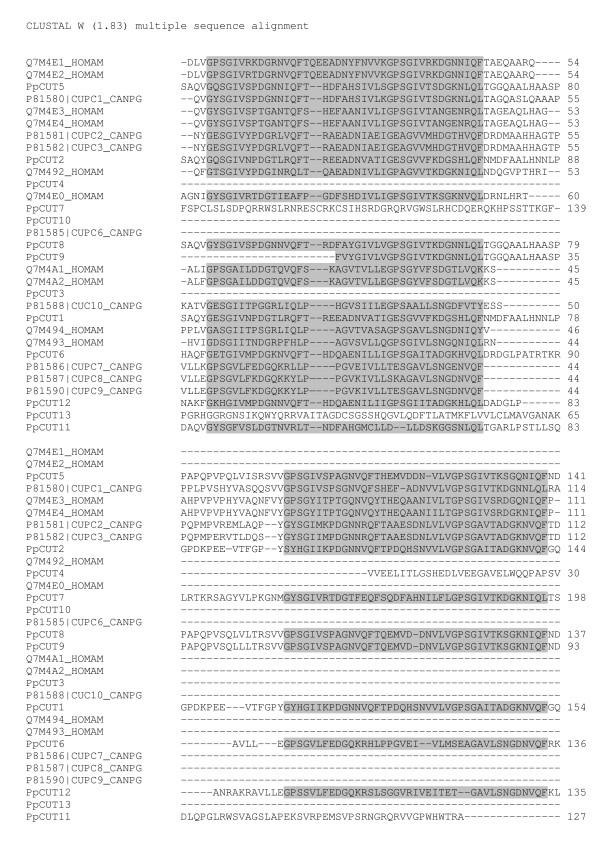
ClustalW alignment of amino acid sequences containing one or more cuticle_1 domain.

**Figure 4:**
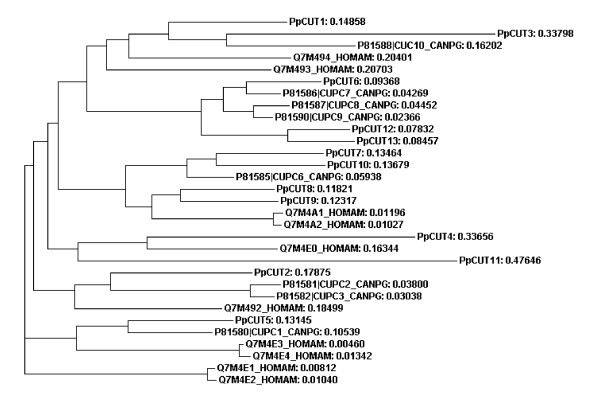
Phylogram of amino acid sequences containing one or more cuticle_1 domain

Proteins with the cuticle_1 domain have previously been isolated from the calcified cuticle of two decapod crustaceans; eight from the crab *Cancer pagurus *[16] and 10 from the lobster *Homarus americanus *[17]. All of the proteins previously isolated contain either 1 or 2 copies of cuticle_1 domains. In our study, transcripts with up to four cuticle_1 domains (see Figure 2) were isolated. Six transcripts containing the cuticle_1 domain isolated in this study have a deduced signal peptide, and four of these also contain a deduced transmembrane region, suggesting that they are secreted across the membrane (Figure 2). The lack of a signal peptide in the other transcripts may be due to an incomplete cDNA sequence, therefore the presence of a signal peptide in all cuticle_1 domain-containing transcripts cannot be ruled out. Each cuticle_1 domain consists of two repeated 15 amino acid motifs with a spacer sequence typically 9 to 12 amino acids in length. The alignment in Figure 3 has been created from the cuticle_1 domain-containing proteins, isolated from *C. pagurus *and *H. americanus*, and from 13 unique differentially expressed cuticle_1 domain-containing transcripts (conceptually translated) isolated in this study. Figure 3 depicts the location of the cuticle_1 domain (denoted by shading). While the cuticle_1 domain is common to all the sequences, many regions outside of this domain do not share homology. Figure 4 is a phylogram of the proteins described above for Figure 3. A phylogram is a branching diagram (tree) assumed to be an estimate of a phylogeny, branch lengths are proportional to the amount of inferred evolutionary change [23]. Figure 4 depicts the hypothesised branching order of the cuticle_1 domain-containing sequences where the branch lengths are proportional to the amount of inferred evolutionary change. The tree suggests that the cuticle_1 domain-containing proteins do not group according to species, implying that these sequences have evolved to have distinct functional roles that are conserved between species. The sequence variability seen in Figure 3 together with the apparent functional grouping viewed in the phylogram, points to a functional variation of the proteins despite their shared domain.

**Figure 5:**
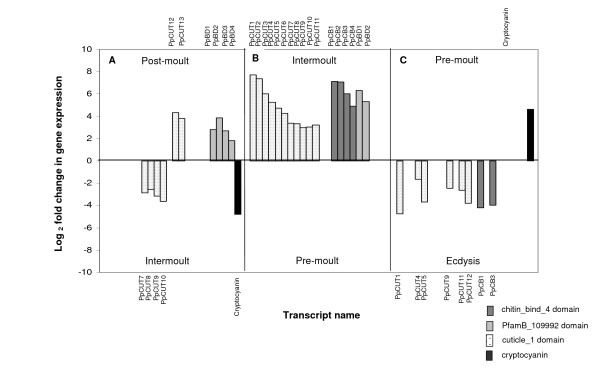
**Differential expression profile of cuticle associated transcripts across moult stages**. Where no bar is evident, no differential gene expression was observed.

Of the 13 *P. pelagicus *transcripts containing the cuticle_1 domain, described above, two transcripts, PpCUT12 and PpCUT13, were up-regulated (eightfold) in the post-moult stage when compared to intermoult. The 11 remaining transcripts, PpCUT1-11, displayed a combined average up-regulation of 9.6-fold in the intermoult stage when compared to crabs in pre-moult. Four of these transcripts, PpCUT7-10, were also down-regulated in post-moult when compared to intermoult by an average of 6.2-fold. Additionally, six transcripts (PpCUT1, 4, 5, 9, 11 and 12) were down-regulated in pre-moult when compared to crabs at ecdysis. No differentially expressed transcripts were detected between the early and late pre-moult stages or between ecdysis and post-moult. For a graphical representation of these data see Table 8 and Figure 5. From these data we can deduce that the up-regulation of many transcripts containing the cuticle_1 domain begins at ecdysis; expression then differentiates into two groups, those transcripts up-regulated in post-moult compared to intermoult and those down-regulated in post-moult compared to intermoult (Figure 5a and Table 8). This converse expression profile of transcripts with the cuticle_1 domain further suggests that a functional and perhaps regulatory difference exists, even between transcripts containing the same domain. The proteins represented by these cuticle_1 domain-containing transcripts may have different mechanisms or modes of action that facilitate their respective roles in exoskeleton formation. High levels of expression of all cuticle_1 domain transcripts (except PpCUT12 and 13) in the intermoult stage compared to early pre-moult (Figure 5b) indicate that formation and/or repair of the exoskeleton continue well into intermoult while expression of these transcripts then decreases dramatically during pre-moult. Multiple proteins containing the cuticle_1 domain have previously been identified in crabs. However, until now, the expression patterns of their corresponding genes across the moult cycle have not been traced. Although the functional role of these proteins, or specifically the cuticle_1 domain, has not been postulated, the domain is thought to be associated with calcification as it has only been identified in proteins isolated from the calcified cuticle of crustaceans [16]. The high level of expression of PpCUT12 and PpCUT13 in post-moult (Figure 5a), and the up-regulation of the other transcripts containing cuticle_1 domains in the intermoult stage (Figure 5b), further support the proposal that proteins with this domain confer moult cycle related changes to the calcified cuticle of crustaceans.

**Figure 6:**
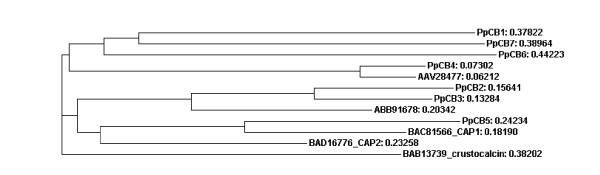
**Phylogram of the alignment of amino acid sequences containing a chitin_bind_4 domain**. Tree distances are shown.

**Figure 7:**
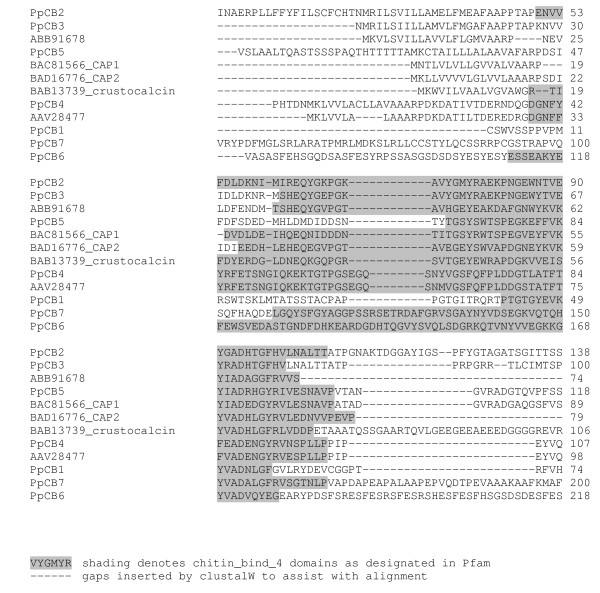
ClustalW alignment of amino acid sequences containing a chitin_bind_4 domain.

**Figure 8:**
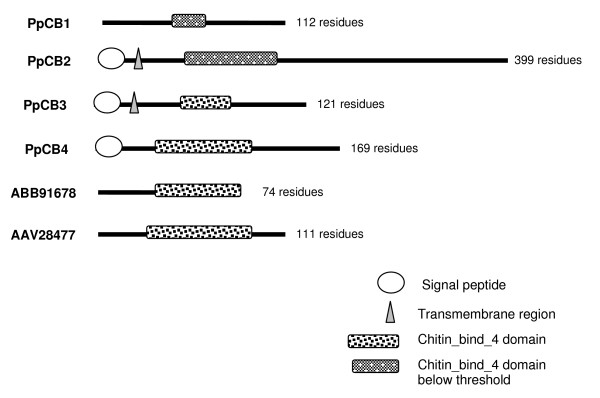
**Schematic diagram of amino acid sequences containing a chitin_bind_4 domain including cuticle proteins previously isolated from *C. sapidus***. Note that the sequences derived from this study (PpCB1-4) may represent partial sequences, the absence of a signal peptide in PpCB1, therefore, may or may not indicate an actual lack of signal peptide. The length of the amino acid sequence is denoted by the number of residues.

Many proteins containing the chitin_bind_4 domain have been isolated from the cuticle of both insects and crustaceans. In addition to their role in chitin binding [15], several crustacean proteins containing this domain also appear to participate in the calcification of the exoskeleton. Calcification associated peptide (CAP) 1 and 2, isolated from the crayfish *Procambarus clarkii*, are multifunctional peptides with anti-calcification, calcium binding and chitin-binding properties [24-26]. Crustocalcin from the prawn *Penaeus japonicus *on the other hand, promotes calcification in addition to having calcium binding properties [27,28]. Despite the commonality of the chitin_bind_4 domain in these proteins, the promotory role of crustocalcin in the calcification process contrasts with the inhibitory properties described for CAP 1 and 2. To investigate the levels of homology between proteins containing the chitin_bind_4 domain, a phylogram (Figure 6) was constructed from the seven (conceptually translated) transcripts isolated in this study (four of these were differentially expressed), CAP 1 and 2, crustocalcin, and two cuticle proteins isolated from the crab *Callinectes sapidus*, all containing the chitin_bind_4 domain. The sequences of the chitin_bind_4 domain-containing proteins appear not to group according to species. Variable regions to either side of the conserved chitin_bind_4 domain are apparent in Figure 7 (the chitin_bind_4 domain is shaded). This points to functional specificity of the sequence outside the domain that is likely to influence the role of the chitin_bind_4 domain in the calcification process. Three of the four differentially expressed transcripts containing chitin_bind_4 domains isolated in this study contained a deduced signal peptide, and two of these also contained a deduced transmembrane region (Figure 8), indicating that they are secreted across the membrane.

Moult cycle related differential gene expression was observed in four unique transcripts containing the chitin_bind_4 domain, PpCB1-4, in this study. These transcripts were highly up-regulated (12-fold) in the intermoult stage when compared against early pre-moult (Figure 5b). We found that only two of these transcripts (PpCB1 and PpCB3) were up-regulated in ecdysis when compared to pre-moult (Figure 5c). The lack of differential expression of transcripts containing the chitin_bind_4 domain between the post-moult and intermoult stages indicates that both stages display similar expression levels of these genes. The up-regulation of chitin_bind_4 transcripts in the intermoult stage compared to pre-moult suggests that these genes are also expressed in post-moult, and that expression is significantly reduced in pre-moult. The high level of expression in intermoult is perhaps unexpected, certainly in terms of exoskeleton formation, which is traditionally associated with the pre- and post-moult stages. However, this expression pattern reflects the expression of other chitin_bind_4 domain-containing genes, CsAMP8.1 and CsAMP6.0 of *C. sapidus*, whereby their expression in arthrodial membranes continued for 32 days post-moult before disappearing [20], apparently due to the late deposition of the un-calcified membranous layer within 16 to 32 days post-moult. The down-regulation of chitin_bind_4 transcripts in the pre-moult stage of the moult cycle when compared with ecdysis, strongly suggest that at least some members of the chitin_bind_4 domain family of proteins are not involved in the synthesis of pre-ecdysial cuticle in crustaceans but rather, are "switched on" at the time of moulting in preparation for the generation of the post-ecdysial, or the hardening of the pre- and post-ecdysial layers. This is consistent with findings for other chitin_bind_4 domain-containing proteins such as CAP 1 and 2, and crustocalcin, which show that expression of their genes occurs in the epidermal tissue only during the post-moult stage [24-28].

**Figure 9:**
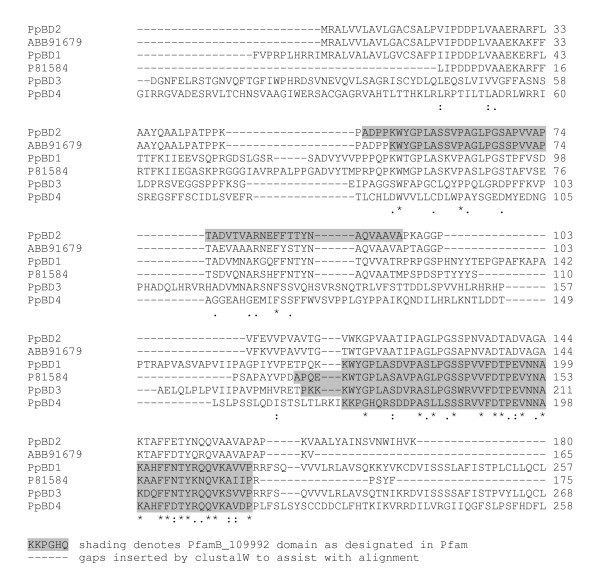
ClustalW alignment of amino acid sequences containing a PfamB_109992 domain.

**Figure 10:**
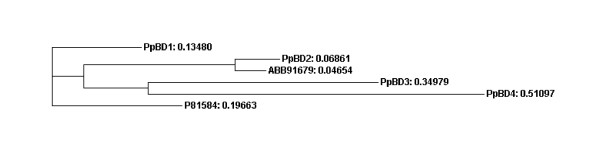
Phylogram of the alignment of amino acid sequences containing a PfamB_109992 domain.

**Figure 11:**
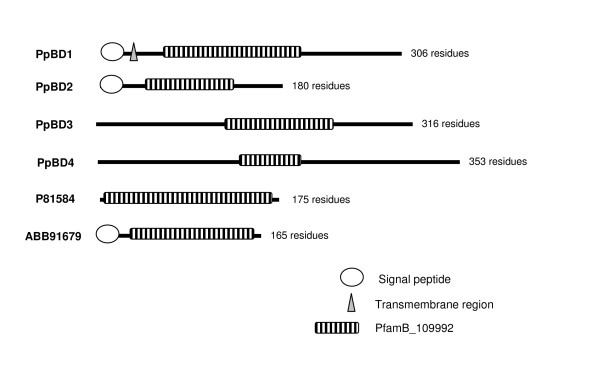
**Schematic diagram of amino acid sequences containing a PfamB_109992 domain, including cuticle proteins previously isolated from *C. pagurus *and *C. sapidus***. Note that the sequences derived from this study (PpPB1-4) may represent partial sequences, the absence of a signal peptide in some transcripts, therefore, may or may not indicate an actual lack of signal peptide. The length of translated amino acid sequence is denoted by the number of residues.

Four transcripts containing the domain PfamB_109992, which as yet remains unannotated, were found to display moult cycle related differential expression profiles in *P. pelagicus*. The PfamB_109992 domain was previously found in a protein isolated from the calcified cuticle of *C. pagurus *(CPCP1876, accession number P81584) [16], and also in the calcified cuticle protein of *C. sapidus *(CP15.0, ABB91679). An alignment of their amino acid sequences (Figure 9), and the phylogram in Figure 10, indicates that they do not group according to species, suggesting that they represent distinct genes, perhaps clustering according to function. The sequence homology may result from conserved functionality. PpBD1 and 2 both contain a deduced signal peptide, while only PpBD1 contains a deduced transmembrane region (Figure 11), suggesting possible secretion of these proteins across the membrane. PpBD1-4 were up-regulated (combined average of 6.4-fold) in the post-moult stage when compared against intermoult (Figure 5a), suggesting that they are relevant to cuticle formation or to changes in the physical properties of the exoskeleton observed after ecdysis. Two of these transcripts, PpBD1 and 2, were also highly up-regulated (by a factor of 12-fold) in the intermoult stage when compared against crabs in the pre-moult period (Figure 5b), signifying their continued expression well into intermoult followed by a decrease in expression during pre-moult. The difference in expression between transcripts containing the PfamB_109992 domain in the intermoult stage when compared to pre-moult is suggestive of a divergence in functionality or regulatory mechanisms. To date, little work has been carried out on proteins containing this domain. However, moult cycle related differential expression evident from the present study, and the previously implied specificity of this domain to the calcified cuticle of crustaceans, signal the importance of proteins carrying this domain to exoskeletal formation.

PpCBM, a transcript sharing sequence homology with a peritrophic membrane chitin binding protein from the cabbage lopper *Trichoplusia ni *(AAR06266), was found to be 6.2-fold up-regulated exclusively in the post-moult stage when compared to intermoult. This transcript contains one chitin binding Peritrophin-A domain (CBM 14) and is found in chitin binding proteins, particularly peritrophic matrix proteins of insects [18]. In insects, these proteins are important determinants for the structural formation and function of the peritrophic membrane, which lines the gut of arthropods and protects it from digestive enzymes [18]. In crustaceans, the mucus layer that coats the undigested products in the stomach and assists in their movement through the digestive tract is also referred to as the peritrophic membrane [29]. Within crustaceans, sequences with the CBM 14 domain have to date only been found in penaeid prawns. The specific up-regulation of the peritrophic membrane chitin binding protein transcript in post-moult, demonstrates its increase in expression during a period associated with cuticle synthesis and hardening. A chitinous portion of the stomach of crabs, and other higher crustaceans, is shed along with the exoskeleton at ecdysis [29], which necessitates the synthesis of a new chitinous matrix in the stomach post-ecdysis. Proteins such as those containing the CBM 14 domain, which has been demonstrated to bind chitin [30], may be involved in the formation and/or structural integrity of the digestive tract.

Moult cycle related differential expression profiles were also observed for three transcripts, PpVER1, PpVER2 and PpVER3, which displayed sequence homology to a LDLa domain containing chitin binding protein from *Drosophila *(Accession number NP_730442), termed *vermiform*. PpVER1 was up-regulated in both the post-moult stage compared to intermoult, and during intermoult when compared to early pre-moult. Whereas PpVER2 was down-regulated in late pre-moult when compared to ecdysis, while PpVER3 was up-regulated in only in the post-moult stage when compared to intermoult. The specific up-regulation of each of these transcripts during various stages of the moult cycle, points to a functional/regulatory difference between these genes, and implicates their involvement in distinct aspects of cuticle formation. *Vermiform*, a gene involved in chitin modification, was found to affect the structural properties of the chitinous matrix of the trachean cuticle of *Drosophila *[31]. The authors demonstrated that this gene is not required for chitin synthesis, secretion, or accumulation but rather for its normal morphology and structure. *Vermiform *codes for a protein with a LDL-receptor ligand binding motif and chitin binding and deacetylation domains. Chitin deacetylase modulates the physical and chemical properties of chitin by deacetylation, which converts chitin into chitosan [32], and may influence the structure and orientation of chitin fibrils in the arthropod cuticle. Luschnig et al [31] demonstrated that *vermiform *is expressed in epidermal cells of *Drosophila*, and that mutations in this gene affect body shape, presumably by altering the structure and rigidity of the epidermal cuticle. Further cuticular abnormalities such as reduced procuticle deposition and aberrant apical membranes were also observed in *Drosophila vermiform *mutants [33]. Proteins with chitin binding properties are important in the structural modifications occurring in the post-ecdysial cuticle. This is the first report of *vermiform *like proteins in crustaceans, and their expression patterns indicate that they have a role in the structural modifications occurring in the post-ecdysial cuticle

In contrast to the cuticle proteins discussed above, cryptocyanin was highly up-regulated in the late pre-moult period (9.2-fold) when compared to crabs in ecdysis, but was down-regulated in post-moult (9.6-fold) when compared to intermoult. This indicates that levels of cryptocyanin transcript decrease at the time of ecdysis and continue to remain low throughout the post-moult period. Cryptocyanin expression then increases in intermoult and remains stable during the pre-moult period. These results concur with those of another study in *C. magister*, in which mRNA levels of cryptocyanin were low immediately post-moult and gradually increased to a peak two thirds into the moult cycle then decreased prior to moulting [14]. The authors demonstrated that similar patterns were exhibited by cryptocyanin protein levels in the hemolymph, where levels remained low in intermoult, increased in pre-moult and dropped dramatically in post-moult [14]. Immunohistochemical and mRNA expression studies in *C. magister*, demonstrated that cryptocyanin is synthesised in the hepatopancreas, secreted into the hemolymph, transferred across the epidermis and incorporated into the extracellular matrix of the new exoskeleton [14]. The converse expression profiles of cryptocyanin and cuticle protein transcripts across the *P. pelagicus *moult cycle observed in this study suggest that they are involved in chronologically different stages of cuticle formation. However this temporal delay is likely due to the different sites of synthesis of each protein, as both protein types play a role in post-ecdysial cuticle formation. The time required between cryptocyanin synthesis and secretion from the hepatopancreas, transportation through the hemolymph and final incorporation into the cuticle, may be responsible for the earlier expression of cryptocyanin mRNA compared with that observed for the other cuticle proteins in this study.

Our study describes the temporal expression patterns of cuticle protein transcripts containing the cuticle_1, chitin_bind_4, PfamB_109992, and CBM14 domains, as well as the hemolymph protein cryptocyanin, across the moult cycle of *P. pelagicus*. Most of the differentially expressed cuticle protein transcripts identified in this study were up-regulated in the intermoult period when compared to the pre-moult. This suggests that synthesis and/or repair of the cuticle occurs well into the intermoult stage of the moult cycle, and that these transcripts are down-regulated in the pre-moult stage and hence not involved in the synthesis of the pre-ecdysial layers of the exoskeleton. Instead these genes may be involved in the synthesis of the post-ecdysial cuticle, or in the exoskeletal hardening process associated with post-moult. None of the cuticle transcripts identified in this study were up-regulated in the pre-moult period, reinforcing the supposition that they are not involved in pre-ecdysial cuticle formation. Only the hemolymph protein cryptocyanin, displayed up-regulation in pre-moult, its involvement in cuticle formation however, also occurs post ecdysis [14].

## Conclusion

Temporal variation in individual transcript expression, both within those transcripts that contain the same domain and between transcripts containing different cuticle domains, was observed in the present study. Wynn and Shafer [20] also demonstrated differential temporal and spatial expression between four genes containing the chitin_bind_4 domain in both calcified and un-calcified cuticle, in *C. sapidus*. This fluctuation in expression levels between cuticle proteins containing the same domain type and the presence of various domains in cuticular proteins indicates that numerous genes are involved in the formation of the crustacean exoskeleton, that they are not up-regulated simultaneously, and that each may have a distinct role in cuticle formation. The moult cycle-related differential expression observed for transcripts containing the same domain type indicates a difference in functionality and perhaps regulation of the corresponding proteins in the crustacean cuticle. We therefore propose that the identification of cuticle proteins be based on the type of domain contained within the protein, rather than the type of cuticle (be it calcified or un-calcified) from which the protein was isolated. As more information becomes available about the role of these domains in the arthropod exoskeleton, the features present within a cuticle protein will become pivotal in describing its function.

Tracing the temporal expression patterns of genes involved in cuticle formation assists in elucidating the mechanisms of cuticle synthesis. Expression information about such genes aids in their functional annotation, as differential gene action underlies the regulation of differential protein accumulation in the cuticle [19]. The expression data presented here provide a chronological depiction of moult cycle related changes to the synthesis of proteins involved in cuticle formation. We have identified a number of candidate genes, by virtue of domain annotation and differential expression data, that could play an important role in the formation and hardening of the crustacean exoskeleton throughout the moult cycle. The expression pattern of these genes, together with conceptual domain annotation, has enabled the discovery of new genes likely to be important to cuticle formation that contain similar motifs to those identified previously. It is evident that cuticle formation and hardening are complex processes involving many components and requiring strict regulatory mechanisms.

## Methods

### Animal selection

*P. pelagicus *crabs were supplied by staff at the Department of Primary Industries and Fisheries (DPI&F) Bribie Island Aquaculture Research Centre (BIARC), Queensland, Australia. The crabs were individually housed in a flowthrough system at an ambient water temperature of 24°C, and fed a commercial diet (Ebistar, Higashimaru, Japan) twice daily. Two size groups of crabs were used, small crabs of an average carapace width of 4 cm, and larger crabs of an average carapace width of 11 cm. All crabs were moult staged by examination of pleopod paddles for epidermal retraction and grouped into the following moult stages; moult (shedding of the exoskeleton), post-moult (pliable exoskeleton), intermoult (hard exoskeleton with no evidence of epidermal retraction) early and late stage pre-moult (based on the extent of epidermal retraction) [34].

### cDNA library construction

Two cDNA libraries were constructed using various source tissues, selected in order to provide a diverse collection of transcripts, and representing a broad range of tissue functions and physiological states in all moult stages. One of the cDNA libraries was synthesised from whole animals in order to obtain transcripts from each tissue type. For this library, six small crabs, from each of the following five moult stages; moult, post-moult, intermoult, early and late pre-moult stages, were selected, snap frozen and ground under liquid nitrogen. The other cDNA library, was derived from organs previously identified as being important to the moult cycle of crustaceans and served to enrich the array with sequences particularly relevant to crustacean moulting. The tissues represented in the *P. pelagicus *organ library were brain, eyestalk, MO and Y-organ. These tissues were obtained from six anaesthetised large *P. pelagicus *crabs from each of moult, post-moult, intermoult, and early and late pre-moult stages, and stored in RNAlater (Ambion, Austin, TX, USA).

Total RNA was purified from each tissue sample using TRIZOL reagent as recommended by the manufacturer (Invitrogen Life Technologies, Carlsbad, CA, USA). Concentration and purity of the RNA were determined using a spectrophotometer (GeneQuant Pro, GE Healthcare UK Ltd., Buckinghamshire, England) with 230, 260 and 280 nm readings. RNA quality was assessed for all samples by visualisation on a denaturing formaldehyde RNA gel (protocol recommended by Qiagen, Valencia, CA, USA) and ethidium bromide staining. Each cDNA library was constructed by pooling equal amounts of total RNA from all moult cycle stages.

A commercial cDNA library synthesis system (SMART cDNA library construction kit, Clontech, Mountain View, CA, USA) was used for the construction of each library according to the manufacturer's instructions. Only the final cloning step was modified so that instead of using the λ TriplEx2 vector supplied with the kit, the size fractionated cDNA was ligated into pGEM-T Easy (Promega, Madison, WI, USA) as per manufacturer's instructions, and transformed into XL10 Gold ultracompetent cells (Stratagene, La Jolla, CA, USA) according to the manufacturer's protocol. A total of 80 clones, randomly selected from each library, were then sequenced and analysed using a BLAST search [35] to determine gene redundancy. The primer used for sequencing was the 5' SMARTlibPCR primer (5' AAGCAGTGGTATCAACGCAGAGT 3') a modification of the SMART IV oligonucleotide supplied with the SMART cDNA library construction kit (Clontech).

### Screening for redundant clones

Upon examination of 160 clones, from the cDNA libraries of both whole crab and crab organ, redundancies for 16S ribosomal RNA were found to be as high as 30%. To remove 16S ribosomal RNA carrying plasmids, all of the clones were first screened for the 16S ribosomal RNA sequence, using a colony hybridisation method [36]. Briefly, three probes, (500, 344 and 300 bp in length) were designed from separate regions of the 16S Ribosomal RNA sequence. These probes were PCR amplified and labelled with P^32^, then hybridised to clones that had been fixed to nitrocellulose filters. Following an overnight incubation at 55°C in hybridisation buffer (6 × SSC and 1% SDS), the filters were washed twice at 55°C in a solution of 6 × SSC and 0.2% SDS for 30 min, sealed within plastic and exposed onto autoradiography films (GE Healthcare UK Ltd.) at -70°C using intensifying screens. The films were then developed according to supplier's instructions.

### Construction of custom *P. pelagicus *cDNA microarrays

A total of 5000 unsequenced clones that had been pre-screened for 16S ribosomal RNA were randomly selected for spotting onto the microarray slides. Of these, 2400 were selected from the whole crab library and 2600 from the crab organ library. These were grown overnight in LB containing 50μg/ml ampicillin. The clones were sent to the AgGenomics microarray printing facility (Bundoora, Victoria, Australia). The clones were PCR amplified using kit supplied primers (Clontech) and contact-spotted (in duplicate) using pins onto amino silane coated glass slides in 50% DMSO buffer. Known crab genes, which were identified at the initial sequencing stage, such as actin (GenBank: EF110528) cryptocyanin (GenBank: EF102021), hemocyanin (GenBank: EF110534), metallothionein (GenBank: EF110529), opsin (GenBank: EF110527) and ubiquitin (GenBank: EF110526) were spotted onto the arrays for use as controls. Genes specifically associated with the moulting process such as moult-inhibiting hormone (MIH) (GenBank: EF110524), crustacean hyperglycaemic hormone (CHH) [GenBank: EF110525] and farnesoic acid methyl transferase (FaMeT) long isoform (GenBank: DQ085282) [37], were isolated separately from *P. pelagicus *through the design of gene specific primers and spotted on to the arrays. In addition universal reference RNA standard controls (Lucidea, GE Healthcare UK Ltd.) were also spotted onto each array, as were negative control spots of 50% DMSO (without cDNA). The cDNA was bound to the slide surface by baking and UV crosslinking.

### Experimental design

In order to identify differential gene expression across moult stages, two consecutive moult stages were compared on each array in a dual colour (Cy3 and Cy5) experiment. RNA samples were pooled across subjects in order to reduce the effect of biological variation. A formula, which dictates the total number of subjects and arrays required for the pooled experiment to obtain gene expression estimates and confidence intervals comparable to those obtained from a non-pooled experiment [38], gave 90% confidence if nine subjects were pooled across a total of three arrays. To this effect, equal amounts of total RNA from three crabs in one moult stage, were pooled, and compared against equal amounts of total RNA pooled from three crabs in another moult stage, on one array. This was repeated three times in total, the different moult stages were labelled with Cy3 or Cy5 respectively. Consecutive moult stages were compared in the following format; post-moult (Cy3) with intermoult (Cy5), intermoult (Cy3) with early pre-moult (Cy5), early pre-moult (Cy3) with late pre-moult (Cy5), late pre-moult (Cy3) with ecdysis (Cy5), and ecdysis (Cy3) with post-moult (Cy5). Figure 1 is a schematic diagram depicting each set of moult stage comparisons.

Technical variation (that is, array-to-array variability) in these microarray experiments was addressed through spot duplication. Two identical grids consisting of each amplified cDNA and including the controls described above were printed onto the left and right sides of each horizontally orientated array, thus affording spatial separation between duplicate spots, to allow for the normalisation of potential hybridisation anomalies.

### Microarray hybridisations

RNA from nine small crabs (six of these were also used in the above-described whole crab cDNA library construction), snap frozen and ground under liquid nitrogen, was isolated using TRIZOL reagent as recommended by the manufacturer (Invitrogen Life Technologies). The RNA was DNase treated using RQ1 RNase free DNase (Promega) as per manufacturer's instructions and purified using RNeasy Mini Kit (Qiagen) as recommended by the manufacturer. RNA quality was assessed by visualisation on a denaturing formaldehyde RNA gel (protocol recommended by Qiagen) using ethidium bromide staining. Concentration and purity of the RNA were determined using a spectrophotometer (GeneQuant Pro) at absorbencies of 230, 260 and 280 nm. A total of 1μg of Lucidea universal RNA control (GE Healthcare) was added to 10μg of pooled total RNA for each moult stage sample, the RNA was converted to cDNA then labelled and hybridised to the array using the 3DNA Array 900 MPX expression array detection kit (Genisphere Inc., Hatfield, PA, USA) according to the manufacturer's protocol. Briefly, RNA was reverse transcribed using a random primer combined with an oligo dT primer. The RNA was then degraded and the cDNA tailed with dTTP followed by ligation to a dendrimer-specific capture oligo (specific for either Cy3 or Cy5). Microarray slides were denatured prior to use by immersion in 95°C MilliQ water for 5 min, the slides were then transferred to 95% ethanol at room temperature for 2 min. Slides were spun dry to reduce streaking at 800 rpm for 2 min. The Cy3 and Cy5 "tagged" cDNAs were combined and then hybridised to the array by overnight incubation in a humidity chamber at 65°C using the kit supplied non-formamide SDS-based buffer and a poly T based blocker, as per manufacturer's specifications. The "tagged" cDNA was washed with a series of three SSC-based buffers, the first wash occurred at 65°C for 15 min, the other wash steps were carried out at room temperature for 10 min each. The slides were spun dry at 800 rpm for 2 min. Fluorescent 3DNA capture reagent (which carries a sequence complementary to the Cy3 and Cy5 tag) was then hybridised to the array using the SDS-based buffer with added Anti-Fade reagent (inhibits photobleaching of Cy5) at 65°C for 4 h. The fluorescent reagent was then washed as described above for the cDNA hybridisation.

### Data analysis

Microarray slides were scanned using a white-light ArrayWorx Biochip Reader (Applied Precision, LLC, Issaquah, Washington, USA). ImaGene (BioDiscovery Inc., El Segundo, CA, USA) was then used to process images and create spot intensity reports, while CloneTracker (Biodiscovery Inc.) was used to generate gene ID mapping files and assign gene identification. Final intensity reports were retrieved as raw spot intensities in tab-delimited files. The data set is deposited in the Gene Expression Omnibus (GEO) database [39] under accession no. GSE6997.

Microarray data analysis was performed by Emphron Informatics (Chapel Hill, Queensland, Australia). Briefly, data was normalised using the robust scatter plot smoother LOESS (also known as "LOWESS" for locally-weighted regression and smoothing scatter plots) [40]. For each chip, normalisation was applied to the left and right sides separately (spatial positioning of clones spotted in duplicate was in the format of two grids located on the left and right side of each array when orientated horizontally). Individual microarray quality was assessed using M vs A scatter plots. M-A plots were constructed for each slide, where the log-intensity ration M = log(Cy3/Cy5) [logCy3-logCy5] were plotted against the mean log-intensity A = [(logCy3+logCy5)/2] as described by [41]. Potential dye intensity biases in the microarray data sets were assessed by examining the back-to-back histograms of Cy3 and Cy5 expression. As each gene is spotted onto an array in duplicate, and three biological replicates are performed per moult stage comparison, a standard error, a *t*-statistic, and *t*-distribution (P value) can be calculated for each gene represented on the array. Standard errors were based on the mean of technical replicates for a given slide. For each of the genes on the slide, a P value for differential expression was calculated using the empirical Bayes procedure [42]. These P values were then adjusted using the false discovery rate (FDR) procedure [43]. This conservative procedure provides control of the family-wise error rate (FWER), which is the probability of at least one false positive. The advantage of controlling the FWER is that any genes identified as differentially expressed are highly likely to be so, however, the disadvantage is that it is easier to omit genes that are differentially expressed. Differential gene expression was only considered significant if the (FDR) adjusted P value was < 0.05. These genes are listed in the results section where M is the *log*_2 _fold change in expression and t is the t-statistic. Positive values of M and t indicate up-regulation in the Cy3 sample whereas negative values of M and t indicate down-regulation in the Cy3 sample.

Genes were considered up- or down-regulated when the logarithm of the gene expression ratio (M) was > 1 or < -1, that is there was a twofold (or greater) difference in expression levels.

### Sequence and phylogenetic analysis

Following hybridisation experiments, clones that displayed differential expression (P ≤ 0.05) patterns across moult stages were sequenced. Overlapping cDNA sequences (contigs), that likely represent the same transcript, and clones without sequence identity to other cDNAs (singlets) were identified by comparing all sequences against one another in sequencher (Gene Codes Corporation, Ann Arbor, MI, USA). The genes were annotated with the name of the highest basic local alignment search tool (BLAST) [44] score from an analysis of GenBank entries by the BLASTx and BLASTn procedures. Amino acid and nucleotide sequence alignments were produced using the ClustalW program [45]. Phylogenetic trees (phylograms) were generated in ClustalW using the Neighbour Joining method [23]. Protein domains were identified from the Pfam database [46], signal peptides were detected using InterProScan [47].

## Competing interests

The author(s) declares that there are no competing interests.

## Authors' contributions

AVK contributed to the conception and design of the project, analysis and interpretation of the data. AVK also carried out the molecular studies and drafted the manuscript. DJM contributed to the design of the project and drafted the manuscript. AE contributed to the conception and design of the project, analysis and interpretation of the data and drafted the manuscript.
